# Squamosamide Derivative FLZ Diminishes Aberrant Mitochondrial Fission by Inhibiting Dynamin-Related Protein 1

**DOI:** 10.3389/fphar.2021.588003

**Published:** 2021-03-19

**Authors:** Hanyu Yang, Lu Wang, Caixia Zang, Xu Yang, Xiuqi Bao, Junmei Shang, Zihong Zhang, Hui Liu, Cheng Ju, Fangyuan Li, Fangyu Yuan, Dan Zhang

**Affiliations:** State Key Laboratory of Bioactive Substrate and Function of Natural Medicine, Department of Pharmacology, Institute of Materia Medica, Chinese Academy of Medical Sciences and Peking Union Medical College, Beijing, China

**Keywords:** mitochondrial dyanmics, DRP1, FLZ, mpp^+^, Parkinson’s disease

## Abstract

Mitochondrial dysfunction is involved in the pathogenesis of Parkinson’s disease (PD). Mitochondrial morphology is dynamic and precisely regulated by mitochondrial fission and fusion machinery. Aberrant mitochondrial fragmentation, which can result in cell death, is controlled by the mitochondrial fission protein, dynamin-related protein 1 (Drp1). Our previous results demonstrated that FLZ could correct mitochondrial dysfunction, but the effect of FLZ on mitochondrial dynamics remain uncharacterized. In this study, we investigated the effect of FLZ and the role of Drp1 on 1-methyl-4-phenylpyridinium (MPP^+^)–induced mitochondrial fission in neurons. We observed that FLZ blocked Drp1, inhibited Drp1 enzyme activity, and reduced excessive mitochondrial fission in cultured neurons. Furthermore, by inhibiting mitochondrial fission and ROS production, FLZ improved mitochondrial integrity and membrane potential, resulting in neuroprotection. FLZ curtailed the reduction of synaptic branches of primary cultured dopaminergic neurons caused by MPP^+^ exposure, reduced abnormal fission, restored normal mitochondrial distribution in neurons, and exhibited protective effects on dopaminergic neurons. The *in vitro* research results were validated using an MPTP-induced PD mouse model. The *in vivo* results revealed that FLZ significantly reduced the mitochondrial translocation of Drp1 in the midbrain of PD mice, which, in turn, reduced the mitochondrial fragmentation in mouse substantia nigra neurons. FLZ also protected dopaminergic neurons in PD mice and increased the dopamine content in the striatum, which improved the motor coordination ability of the mice. These findings elucidate this newly discovered mechanism through which FLZ produces neuroprotection in PD.

**Figure:**
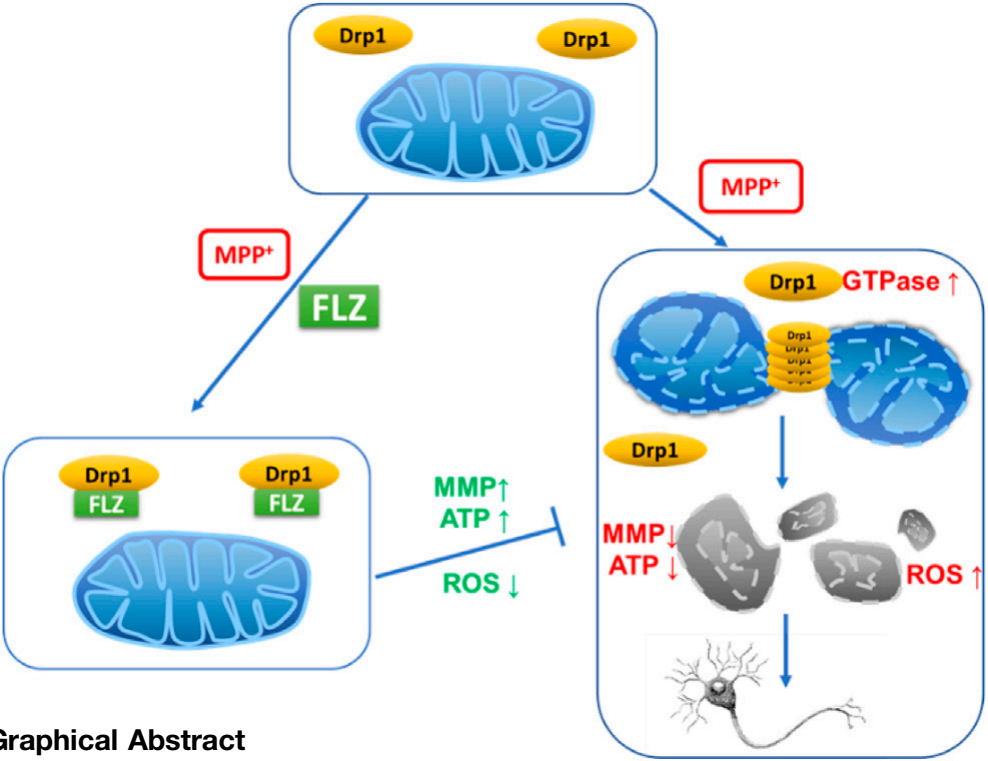
**Graphical Abstract**

## Highlights

FLZ blocked Drp1, inhibited Drp1 enzyme activity, reduced excessive mitochondrial fission in cultured neurons.

FLZ improved mitochondrial integrity and membrane potential by inhibiting mitochondrial fission and ROS production, resulting in neuroprotection.

FLZ exhibited protective effects on dopaminergic neurons.

FLZ reduced excessive mitochondrial fission in MPTP-induced PD mouse model.

## Introduction

Mitochondria are the primary organelles involved in the production of cellular erobic respiration. They provide the necessary energy needed by cells through the oxidative phosphorylation of cellular respiration to produce ATP. Mitochondria are highly dynamic organelles that continuously undergo fission and fusion cycles (McBride et al., 2006). The two opposing processes of fission and fusion are critical events in numerous physiological processes, including programmed cell death, calcium homeostasis, autophagy, redox signaling, and innate immunity (Suliman and Piantadosi, 2016; Smith et al., 2012). Normal mitochondrial dynamics are a balance of mitochondrial division and fusion that affects the number, size, and location of mitochondria to meet the needs of the physiological activities occurring in cells. Due to the essential role of dynamic mitochondrial homeostasis in eukaryotic cells in maintaining normal cell homeostasis, considerable research has focused on the role of mitochondrial dynamics in metabolic, cardiovascular, neoplastic, and neurodegenerative diseases.

Five essential proteins primarily control mitochondrial fission and fusion. Of these five proteins, dynamin-related protein 1 (Drp1) and mitochondrial fission protein 1 (Fis1) are responsible for mitochondrial fission, while mitofusin 1/2 (Mfn1/2) and optic atrophy 1 (Opa1) play critical roles in mitochondrial fusion (Flippo and Strack, 2017). During the fission process, Drp1 translocates from the cytoplasm to the outer mitochondrial membrane to respond to cellular stimuli (Chang and Blackstone, 2010), At that location, Drp1 forms a ring-shaped structure that wraps around the mitochondria in a GTPase-dependent manner (Smirnova et al., 2001). Over-activation of Drp1 can result in excessive mitochondrial fragmentation, which disrupts calcium homeostasis, decreases ATP production, increases reactive oxygen species (ROS) production, and eventually results in cell death (Qi et al., 2018). Inhibition of Drp1 using RNA interference or Drp1-dominant-negative mutations resulted in reduced mitochondrial fragmentation and improved mitochondrial function (Ruiz et al., 2018; Aouacheria et al., 2017; Filichia et al., 2016). Thus, these studies demonstrated that Drp1 is crucial in regulating mitochondrial fission and maintaining mitochondrial homeostasis.

Parkinson’s disease (PD) is one of the most prevalent neurodegenerative diseases worldwide. Several factors contribute to the pathogenesis of PD, of which mitochondrial dysfunction plays an important role in both sporadic and familial forms of the disease (Ahmad et al., 2013). Strict regulation of fission and fusion processes is necessary to maintain the normal metabolic activity of mitochondria (Bose and Beal, 2016). Researchers have reported marked differences between the mitochondrial network morphology in fibroblasts from PD patients compared to control patients; specifically, PD patients exhibit increased fragmentation of the mitochondrial network, and the degree of mitochondrial branching was significantly decreased compared with control patients (Haylett et al., 2016; Burbulla et al., 2010). Various PD‐related neurotoxic molecules, such as MPP^+^ and 6-hydroxydopamine, increased mitochondrial fission *in vitro* by upregulating Drp1 (Chuang et al., 2016; Gomez-Lazaro et al., 2008; Qi et al., 2013). Mutations in PD-related genes, including parkin, DJ-1, PINK1, and alpha-synuclein, lead to mitochondrial fission, resulting in neurons that are more susceptible to dysfunction and death (Büeler, 2009; Wang et al., 2012; Wang et al., 2011a; Nakamura et al., 2011). Notably, excessive mitochondrial fragmentation induced by high Drp1 levels can be reversed by a dominant-negative Drp1 mutation (Wang et al., 2011b). Recent studies have shown that Drp1-induced aberrant mitochondrial fission plays a vital role in dopaminergic cell apoptosis in PD (Rappold et al., 2014). Enhancement of Drp1 promotes mitochondrial fission and PD-associated dopaminergic nerve cell apoptosis, whereas Drp1 inhibition reverses aberrant mitochondrial fission, reduces nerve cell apoptosis, and improves symptoms of PD (Zhang et al., 2019). These results suggest that Drp1 might be the key molecule in regulating mitochondrial fission and function in PD pathogenesis. Therefore, it is of great importance to find Drp1 inhibitors to develop new therapies for PD.

**FIGURE 1:**
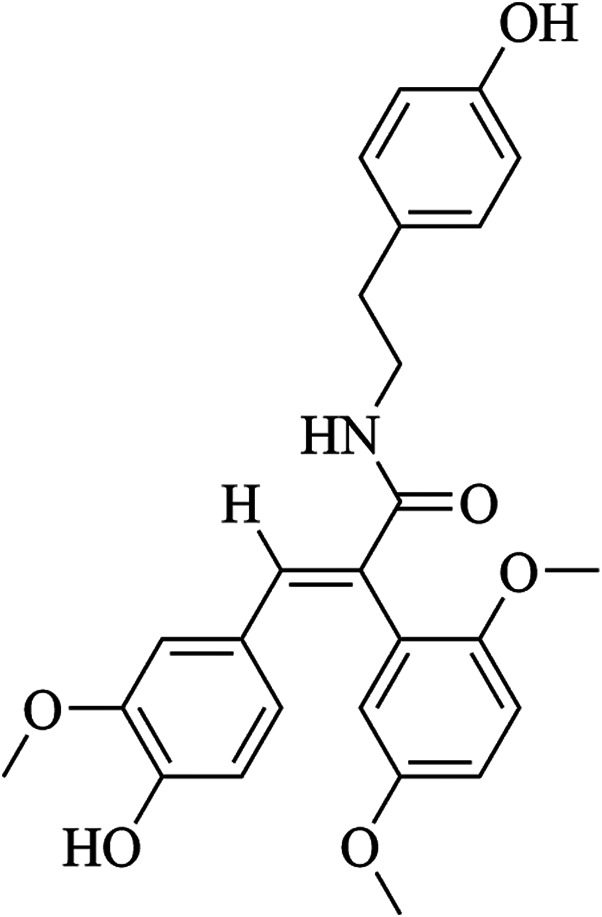
Chemical structure of FLZ.

FLZ, which is formulated as N-[2-(4-hydroxy-phenyl)-ethyl]-2-(2,5-dimethoxy-phenyl)-3- (3-methoxy-4-hydroxy-phenyl)-acrylamide, is a novel synthetic cyclic analogue of natural squamosamide Figure 1. FLZ is currently in phase I clinical trials, and the therapeutic potential of FLZ in treating PD has been studied extensively in a range of PD animal models (Tai et al., 2013; Bao et al., 2017). Primary mechanistic studies have demonstrated that FLZ exerts protective effects on injured neurons via normalization of mitochondrial function and inhibition of apoptosis; however, the precise mechanisms involved are still unknown (Ye et al., 2014). Given the important role of Drp1 in regulating mitochondrial dynamics, we designed a series of experiments to investigate and confirm the role and mechanisms of Drp1 in the therapeutic effects of FLZ on PD. Our *in vitro* studies were verified using a subacute PD mouse model induced using MPTP, which confirmed the molecular mechanism of Drp1 in FLZ treatment of PD.

## Materials and Methods

### SH-SY5Y and Hela Cell Culture

SH-SY5Y cell line and Hela cell line were purchased from the Cell Culture Center at the Institute of Basic Medical Sciences, Chinese Academy of Medical Sciences and Peking Union Medical College. Cells were cultured in Dulbecco’s Modified Eagle Medium (DMEM, Solarbio, China) supplemented with 10% heat-inactivated fetal bovine serum (FBS, Sijiqing, China).

### Primary Dopaminergic Neuron Culture

For primary dopaminergic neuron culture, midbrains from embryonic day 14 (E14) Sprague–Dawley rats were used according to the methods previously reported (Gaven et al., 2014). Briefly, the midbrains of embryos were dissected into Hanks Balanced Salt Solution (HBSS) on ice and cut into small pieces. Cells were dissociated with 1% DNase I and 2.5% trypsin in HBSS at 37°C. The cell suspension was filtered through a 70 μm cell strainer and seeded on poly-d-lysine coated (Cat#P1399, Sigma, United States) coverslips at a density of 5 × 10^5^ cells per well and maintained in DMEM/F12 (Cat#SH30023, HyClone, United States) with 10% FBS (Cat#16000–044, Gibco, United States), 1% Glutamax (Cat#35050–061, Gibco, United States) and 1% penicillin–streptomycin for 4 h, and then switched to neurobasal medium (Gibco, United States) supplemented with 2% B27 and 1% Glutamax for more than 10 days. The media were changed every other day.

### Development of PD Mouse Model and Treatment

Male C57/BL6 mice (8 weeks old, 18–22 g) were supplied by the Animal Center of Chinese Academy of Medical Sciences (Beijing, China). Mice were maintained in a 12/12 light/dark cycle at 24°C and received food and water ad libitum. All protocols and procedures involving animals were approved by the Animal Care and Welfare Committee of Institute of Materia Medica, Chinese Academy of Medical Sciences and Peking Union Medical College.

The subacute PD mouse model was developed according to previously described methods (Wang et al., 2016). Mice were treated with vehicle (0.5% CMC-Na, p.o.) or FLZ (75 mg/kg, suspended in 0.5% CMC-Na, p.o.) 30 min before each MPTP hydrochloride injection (Sigma, Aldrich, United States; 30 mg/kg, i. p.) for seven consecutive days. From day 8 to day 12, mice were only treated with vehicle or FLZ. At day 12, motor behaviors were accessed by rotarod test and pole test.

### Rotarod Test

The rotarod test is widely used to evaluate animals’ balance and coordination on a rotating beam. In this study, the rotarod test was performed as previously described (Wang et al., 2016). The mice were positioned on the rotarod, and the rotarod was set to revolve at 30 rpm for up to 120 s. The latency represented the first time that mice fell off the rod and that time could be automatically recorded by the rotarod. Researchers were blinded to the group assignment performed the behavioral tests.

### Pole Test

The pole test was used to demonstrate bradykinesia in PD mice. The mouse was placed head-downward on the top of a vertical rough-surfaced pole (diameter 8 mm; height 50 cm) and the time until it descended to the floor was recorded with a maximum duration of 30 s. If the mouse did not descend within 30 s, it was guided. The mice were pretrained before experimentation. Each animal performed two trials with an interval of 1 h.

### Striatal Dopamine Analysis

Mouse striatal dopamine was analyzed by high-performance liquid chromatography with fluorimetric detection (HPLC-FD). Each striatum was weighed and suspended in 0.6 M perchloric acid (W/V = 1:30). Samples were grinded and then centrifuged. Potassium dihydrogen phosphate solution (20 mM potassium citrate, 2 mM EDTA Na2, 300 mM potassium dihydrogen phosphate) was added to the supernatant and centrifuged twice. An aliquot of the supernatant was separated in 20 min on a reversed-phase column (C18, 5 μm, 150 × 2.0 mm) with acetate buffer (85 mM Citric Acid, 100 mM Sodium acetate anhydrous, 0.2 mM EDTA Na2, pH 3.68)–methanol (85:15, v/v) as mobile phase; the flow rate was 1 ml/min. The fluorescence measurements were carried out at 320 nm with excitation at 279 nm.

### Transfection With Plasmid of Drp1

Plasmid encoded Drp1 gene was obtained from Genecopoeia Biotechnology (China). Adhered Hela cells at 70% confluency were transfected for 24 h with Drp1 plasmid using Lipofectamine 3,000 (Invitrogen, United States) according to the manufacturer's instructions.

### Isolation of Mitochondrial-Enriched Fraction

Mitochondrial-enriched fraction was separated by Mitochondria Isolation Kit (Applygen, China) according to the user guides. Briefly, after washing cell or mouse brain tissue with cold PBS, the cell or tissue was grinded in a Dounce Grinder with cold Mito Solution. Then the homogenates were centrifuged at 800 g for 5 min at 4°C, after that we discarded the pellets and centrifuged the supernatants at 10,000 g for 10 min at 4°C. The pellets were washed with Mito Solution and centrifuged again. The final pellets were suspended in Mito Solution.

### Immunocytochemistry

Cells cultured on coverslips were washed by PBS for 3 times, then incubated with 100 nM MitoTracker® Orange CMTMRos (Invitrogen, United States) at 37°C for 30 min, then the cells were fixed in 4% formaldehyde for 15 min. After that we added 0.1% Triton X-100 to permeabilize the cells. After incubation with 3% normal goat serum for 2 h at room temperature, fixed cells were incubated overnight at 4 C with antibodies against Drp1 (1:100), *β*-tubulin (1:100) or TH (1:100). Cells were washed with PBS and then incubated for 2 h with Goat Anti-Rabbit IgG H&L (Alexa Fluor® 488) Antibody (1:500, Abcam, United States) followed by incubation with DAPI for 10 min. Coverslips were imaged by confocal microscopy (Leica, Germany). Cells shown a filamentous mitochondrial network were classified as normal. Cells with fragmented mitochondria were classified as fragmented.

### Immunohistochemistry Assay

Mice were perfused with 0.9% NaCl, and the brains were dissected and post fixated with 4% paraformaldehyde for 1 day. Then brains were cut coronally into 30 μm thick sections. The sections were permeabilized and then blocked with 10% donkey serum containing 0.3% Triton X-100 for 0.5 h. Subsequently, the sections were incubated overnight with TH primary antibody (1:50) at 4°C, and then they were incubated with secondary antibody conjugated with streptavidin-labeled peroxidase followed by DAB visualization. The sections were mounted on glass microscope slides, and the stained sections were scanned using a Pannoramic MIDI Digital Slide Scanner (3DHISTECH, Hungary). The numbers of positive cells were calculated using ImageJ software according to the manual. At least four sections were analyzed in each group.

### GTPase Activity Assay

100 ng Drp1 recombinant protein (Abnova, China) were incubated with or without FLZ for 30 min, respectively. The GTPase activity was measured using a GTPase ELIPA Biochem Kit (Cytoskeleton, United States) following the manual. To measure the GTPase activity of Drp1 in SH-SY5Y cells, 1 × 10^6^ cells were extracted and immunoprecipitated with 2 μl anti-Drp1 antibodies and 10 μl Protein A/G agarose overnight at 4 C. Then the GTPase activity was measured using the GTPase assay kit according to the manual.

### qPCR Assay

Total RNA was isolated using a TransZol Up Plus RNA Kit (Transgen, China) and cDNA was synthesized from 1 mg of total RNA using a TransScript One-Step gDNA Removal and cDNA Synthesis SuperMix kit (Transgen, China) according to the manufacturer’s instructions. qRT-PCR was performed using a TransStart Tip Green qPCR SuperMix (Transgen, China) and ABI7900 instrument (Applied Biosystems, United States). The detected expression of mRNA was normalized using *β*-ACTIN as an internal control. The relative mRNA levels were calculated by the 2^−ΔΔCt^ method. Prime sequences used in the study were shown in Table 1.

**TABLE 1:** Prime sequences.

Gene	Primer sequence (5′-3′)		
	Forward	Reverse
Drp1	AAT CCT AAT TCC ATT ATC CTC GCT	ACC AGT AGC ATT TCT AAT GGC	
Fis1	GTC CAA GAG CAC GCA GTT TG	ATG CCT TTA CGG ATG TCA TCA TT
Opa1	TGT GAG GTC TGC CAG TCT TTA	TGT CCT TAA TTG GGG TCG TTG
Mfn1	TGG CTA AGA AGG CGA TTA CTG C	TCT CCG AGA TAG CAC CTC ACC
Mfn2	CTC TCG ATG CAA CTC TAT CGT C	TCC TGT ACG TGT CTT CAA GGA A
β-ACTIN	GCA CCA CAC CTT CTA CAA	TAC GAC CAG AGG CAT ACA

### Measurements of Mitochondrial Function in Culture Cells

Cells were cultured in black 96-well plates at a density of 8 × 10^3^ cells/well and treated with FLZ (10 μM) for 30 min followed by exposed to MPP^+^ (2 mM) for 4 h. After that, cells were stained with CM-H2DCFDA (Applygen, China, for ROS) or JC-1(Beyotime, China, for MMP) at 37°C. Cells were washed by PBS for three times. A fluorescence microplate reader was used for the detection according to manufacturer’s instructions. For ATP concentration detection, cells were cultured in 6-well plates at a density of 4 × 10^6^ cells/well and treated with FLZ (10 μM) for 30 min followed by exposed to MPP^+^ (2 mM) for 24 h. ATP concentration were measured using Enhanced ATP assay Kit (Beyotime, China) following the instructions.

### Measurements of Mitochondrial Function in Isolated Mouse Brain Mitochondria

100 μl isolated mouse brain mitochondria (1 mg protein/ml) were incubated with 100 ng recombinant Drp1 protein with or without FLZ (10 μM) for 20 min at 37°C. After incubation, ROS level, mitochondrial membrane potential and ATP concentration were measured using the kits as above according to the manual.

### Western Blot Analysis

The cells were dissected in RIPA lysate buffer with protease phosphatase inhibitor and protease inhibitor. The total protein concentrations were determined by BCA kit (Applygen, China) to ensure equal sample loading. Protein contents were separated on SDS-poly-acrylamide gels (10%) and then transferred into a 0.45 μm polyvinylidene fluoride membrane (Millipore, United States) which were blocked with 5% skim milk-TBST (20 mM Tris HCl, pH 7.5, 500 mM NaCl, 0.1% Tween 20) for 1 h. The membranes were probed with the following antibodies: *β*-ACTIN (1:10,000), Drp1 (1:1,000), p-Drp1 (Ser616) (1:1,000), GAPDH (1:1,000), TH (1:1,000), overnight at 4°C, and then incubated with secondary antibody (1:2,000, Abclonal, China) for 2 at room temperature. The blots were visualized by incubating the membranes with ECL Plus reagents (Yeasen, China) and the images were recorded by LAS-4000 chemiluminescence system (GE Healthcare, United States). The blot densities were assessed by Gel-pro analyzer 4.0.

### Molecular Docking

The binding mode of FLZ and Drp1 was investigated by a docking simulation using CDocker program in Discovery Studio 2018. The X-ray crystal structure of Drp1 (PDB-ID: 4H1V) was obtained from the protein data bank (http://www.rcsb.org). The protein is subjected to dehydration, hydrogenation, loop region refinement, and the binding site is selected from natural ligand in the eutectic complex. The natural ligand was prepared and then re-dock to the active site, with default parameters. The RMSD value between the docking conformation which has the highest CDocker energy and the original ligand conformation was 2.18, which was smaller than the crystal resolution of 2.3, proving the reliability of the docking method. Then, we docked FLZ into the active site of Drp1 with the same parameters.

### Transmission Electron Microscopy

The mouse brains were fixed in 100 μM sodium cacodylate buffer (pH 7.2) that contained 2.5% glutaraldehyde, 1.6% paraformaldehyde, 0.064% picric acid, and 0.1% ruthenium red. The brains were gently washed and post-fixed for 1 h in 1% osmium tetroxide plus 0.8% potassium ferricyanide, in 100 mM sodium cacodylate, pH 7.2. After thorough rinsing in water, the tissues were dehydrated using a graded series of ethanols followed by acetone; they were infiltrated overnight in 1:1 acetone:Epon 812 and infiltrated for 1 h with 100% Epon 812 resin. The tissues were embedded in Epon 812 resin. After polymerization, 60–80 nm thin sections were cut using a Reichert ultramicrotome (1680 Campus Delivery, Fort Collins, Colorado, United States) and stained for 5 min in lead citrate. They were rinsed and post-stained for 30 min in uranyl acetate and then were rinsed again and dried. Electron microscopy was performed at 60 kV using a Morgagni Philips TEM (1680 Campus Delivery, Fort Collins, Colorado, United States) equipped with a charge-coupled device (CCD).

### Statistical Analysis

Data are expressed as the means ± SEM (standard error of the mean). Statistically significant differences were performed by the Statistical Package for GraphPad Prism 6.0 software using one-way ANOVA with appropriate post hoc testing. *p* < 0.05 was considered to be statistically significant.

## Results

### FLZ Reduced MPP^+^-Induced Mitochondrial Fragmentation and Abnormal Mitochondrial Distribution

**FIGURE 2:**
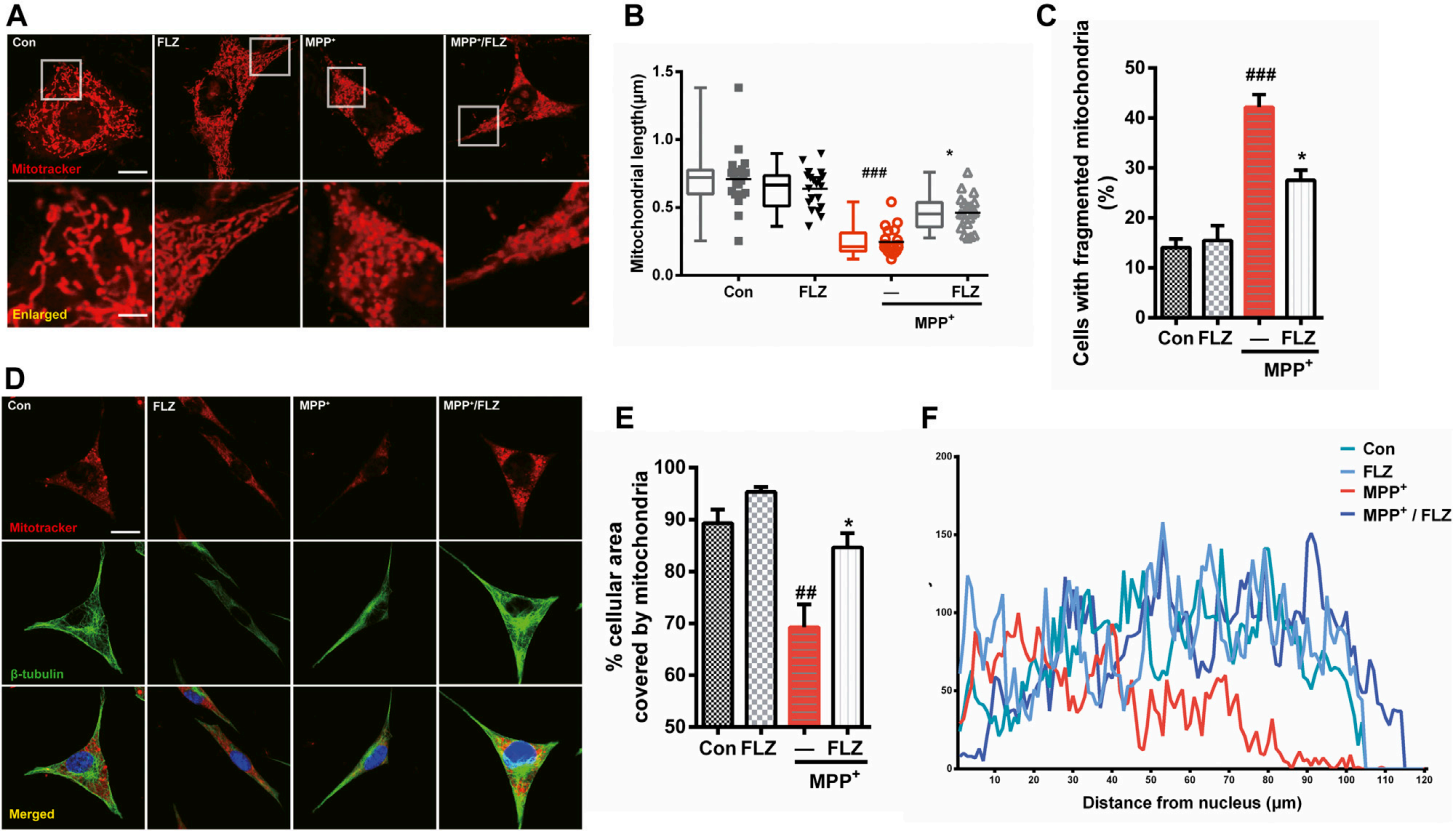
FLZ treatment reduced MPP^+^-induced mitochondrial fragmentation and abnormal mitochondrial distribution. Cultured SH-SY5Y cells were treated with FLZ (10 μM) for 30 min and then exposed to MPP^+^ (2 mM) for 4 h **(A)** Mitochondrial morphology was analyzed by a confocal microscope. Cells were stained with mitotracker (red) and DAPI (blue) (scale bar: up:15 μm, down: 3 μm). **(B)** Statistical analysis of mitochondrial length was measured by Image-pro Plus software. Data are presented as the means ± SEM from 4 independent experiments of 20–30 random selected cells **(C)** The percentage of cells with fragmented mitochondria relative to the total number of cells was counted by an observer blinded to the experimental conditions. At least 200 cells per group were counted. **(D)** Mitochondrial distribution was analyzed by a confocal microscope. Cells were stained with mitotracker (red) and DAPI (blue) (scale bar: 20 μm). **(E)** Statistical analysis of the percentage of cellular area covered by mitochondria were measured by ImageJ Software. Data are presented as the means ± SEM from 4 independent experiments of 20–30 random selected cells **(F)** Shown are representative data of mitochondria (red fluorescence) distribution. One-way ANOVA followed by Tukey post hoc comparisons tests were performed in all statistical analyses. ^##^ *p* < 0.01 and ^###^ *p* < 0.001 vs. Control group, ^*^ *p* < 0.05 vs. MPP^+^ treated group.

The primary manifestation of abnormal mitochondrial dynamics is excessive mitochondrial fragmentation and altered mitochondrial distribution. Therefore, we assessed the effect of FLZ on the mitochondrial morphology and distribution in MPP^+^-treated SH-SY5Y cells to confirm its protective effects on mitochondria. We observed severe mitochondrial fragmentation in cultured cells that were exposed to MPP^+^. The mitochondria in treated cells appeared punctate or dot-like. After FLZ treatment, the mitochondria lengths were markedly increased (Figures 2A,B, *p* = 0.0207 vs. MPP^+^ treated group). The number of cells with fragmented mitochondria decreased when the MPP^+^-treated cells were incubated with FLZ (Figure 2C, *p* = 0.0104 vs. MPP^+^ treated group). Excessive mitochondrial fragmentation often led to the accumulation of mitochondria in the perinuclear area (Trevisan et al., 2018), It was noted that FLZ treatment significantly increased the cellular area occupied by mitochondria (Figures 2D–F, *p* = 0.0253 vs. MPP^+^ treated group). Importantly, exposure to FLZ alone did not affect mitochondrial dynamics in normal cells. These results indicated that FLZ treatment could improve mitochondrial dynamics by alleviating abnormal mitochondrial fragmentation and correcting abnormal mitochondria distribution in neurons challenged with MPP^+^.

### FLZ Reduced MPP^+^-Induced Mitochondrial Fragmentation via Regulation of Drp1.

**FIGURE 3:**
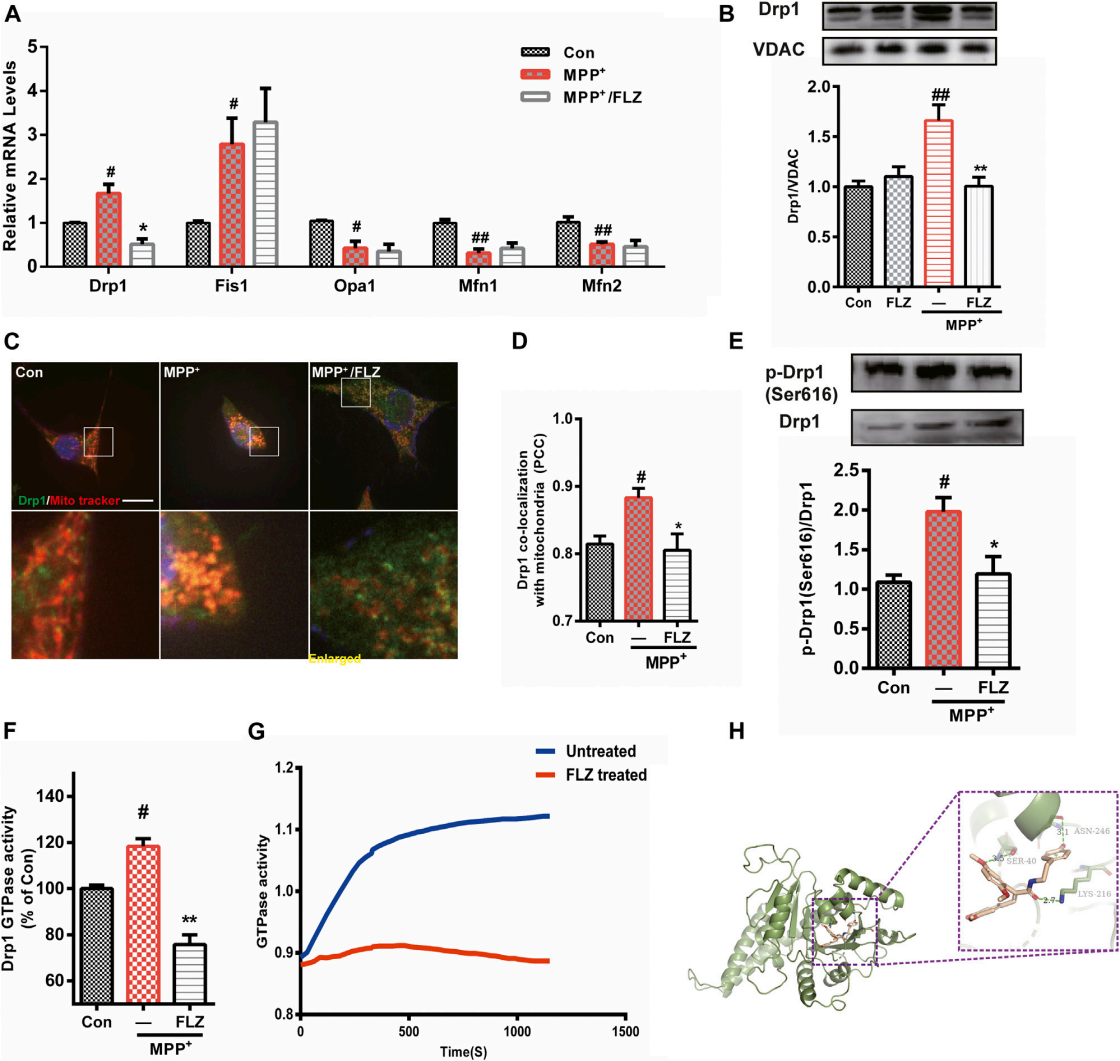
FLZ treatment reduced Drp1 association with mitochondria and decreased Drp1 GTPase activity. Cultured SH-SY5Y cells were treated with FLZ (10 μM) for 30 min followed by incubation with MPP^+^ (2 mM) for 4 h **(A)** mRNA expression of mitochondrial dynamic associated proteins were analyzed by qRT-PCR. **(B)** Western blot analysis of mitochondrial fractions of cells was determined by Drp1 and VDAC antibodies. VDAC was used as a loading control **(C)** Drp1/mitochondria colocalization was determined by confocal analysis. Cells were stained with Drp1 antibody (green), mitotracker (red) and DAPI (blue). Lower panels show enlarged areas of the white boxes in the above panels (scale bar: up: 20 μm, down: 4 μm). **(D)** Pearson’s coefficient per cell of Drp1/mitochondria colocalization was determined using Image-pro Plus software and is provided as a histogram **(E)** p-Drp1 (Ser616) level of SH-SY5Y cells was measured by western blot analysis with p-Drp1 (Ser616) and Drp1 antibodies **(F)** The GTPase activity of immunoprecipitated Drp1 from cultured cells was measured by the Enzyme Linked Inorganic Phosphate Assay. **(G)** The GTPase activity of Drp1 recombinant protein was measured by the Enzyme Linked Inorganic Phosphate Assay. 100 ng Drp1 recombinant protein were incubated with or without FLZ. Shown are representative data out of 4 independent experiments. **(H)** Docked molecular model of FLZ in the active site of the Drp1 (PDB ID: 4H1V). One-way ANOVA followed by Tukey post hoc comparisons tests were performed in all statistical analyses. ^#^ *p* < 0.05 and ^##^ *p* < 0.01 vs. Control group, ^*^ *p* < 0.05 and ^**^ *p* < 0.01 vs. MPP^+^ treated group.

The balance of mitochondrial fission and fusion contributes to normal mitochondrial function. Since FLZ treatment reduced MPP^+^-induced mitochondrial fragmentation, we determined whether this phenomenon was caused by the inhibition of mitochondria fission or the promotion of mitochondria fusion. We investigated the effects of FLZ in MPP^+^-exposed SH-SY5Y cells on Drp1, Fis1, Opa1, and Mfn1/2, which are the primary proteins that regulate mitochondrial dynamics. We determined that the mRNA levels of the fission genes, Drp1 and Fis1, were significantly increased by exposure to MPP^+^, and the mRNA levels of the fusion genes, Opa1 and Mfn1/2, were substantially reduced. These results demonstrated that FLZ could decrease the mRNA level of Drp1 and not affect the other genes (Figure 3A). This evidence indicated that FLZ likely reduced MPP^+^-induced mitochondrial fragmentation through inhibition of aberrant mitochondrial fission by regulating Drp1 expression. Therefore, we focused on Drp1 to assess its role in mitochondrial fission regulation through exposure to FLZ.

Drp1 is a critical molecule involved in the regulation of mitochondrial fission, and its translocation from the cytosol to mitochondria is essential in activating the process of fission (Chang and Blackstone, 2010). FLZ treatment dramatically inhibited Drp1 translocation from the cytosol to mitochondria in MPP^+^-treated cells (Figure 3B, *p* = 0.0027 vs. MPP^+^ treated group). Confocal imaging analysis showed that FLZ incubation decreased the co-localization of Drp1 and mitochondria induced by MPP^+^ (Figures 3C,D, *p* = 0.0375 vs. MPP^+^ treated group).

The recruitment of Drp1 to mitochondria is strictly regulated by several post-translational modifications, of which, phosphorylation has been studied extensively (Otera et al., 2013). As shown in Figure 3E, FLZ markedly decreased Drp1 phosphorylation at Ser616 in SH-SY5Y cells exposed to MPP^+^(*p* = 0.0414 vs. MPP^+^ treated group). Since Drp1 is a small GTPase, it can wrap around mitochondria in a GTPase-dependent manner. We observed that FLZ treatment eliminated the increased Drp1 GTPase activity that occurred in MPP^+^-treated SH-SY5Y cells (Figure 3F, *p* = 0.0075 vs. MPP^+^ treated group). FLZ also directly inhibited the GTPase activity of recombinant Drp1 protein (Figure 3G).

We used molecular docking to verify the interaction of FLZ with Drp1. The preferred coordination mode of FLZ with Drp1 is shown in Figure 3H. From our analysis of the binding interactions, we determined that FLZ interacted with Ser40 amino acid residues in the phosphate-binding loop of Drp1 via hydrogen binding interactions (the hydrogen bond distance is 3.5 Å). FLZ also interacted with Lys216 amino acid residues in the G4 element, and Asn246 amino acid residues in the G5 element, via hydrogen binding interactions (the hydrogen bond distance is 2.7 Å and 3.1 Å, respectively). Thus, these results indicated that FLZ directly binds to the active site of Drp1, and reduced Drp1 recruitment to mitochondria. This binding also decreased Drp1 GTPase activity, which resulted in diminished mitochondria fragmentation.

### FLZ Reduced Mitochondrial Fragmentation in Cells Overexpressing Drp1

**FIGURE 4:**
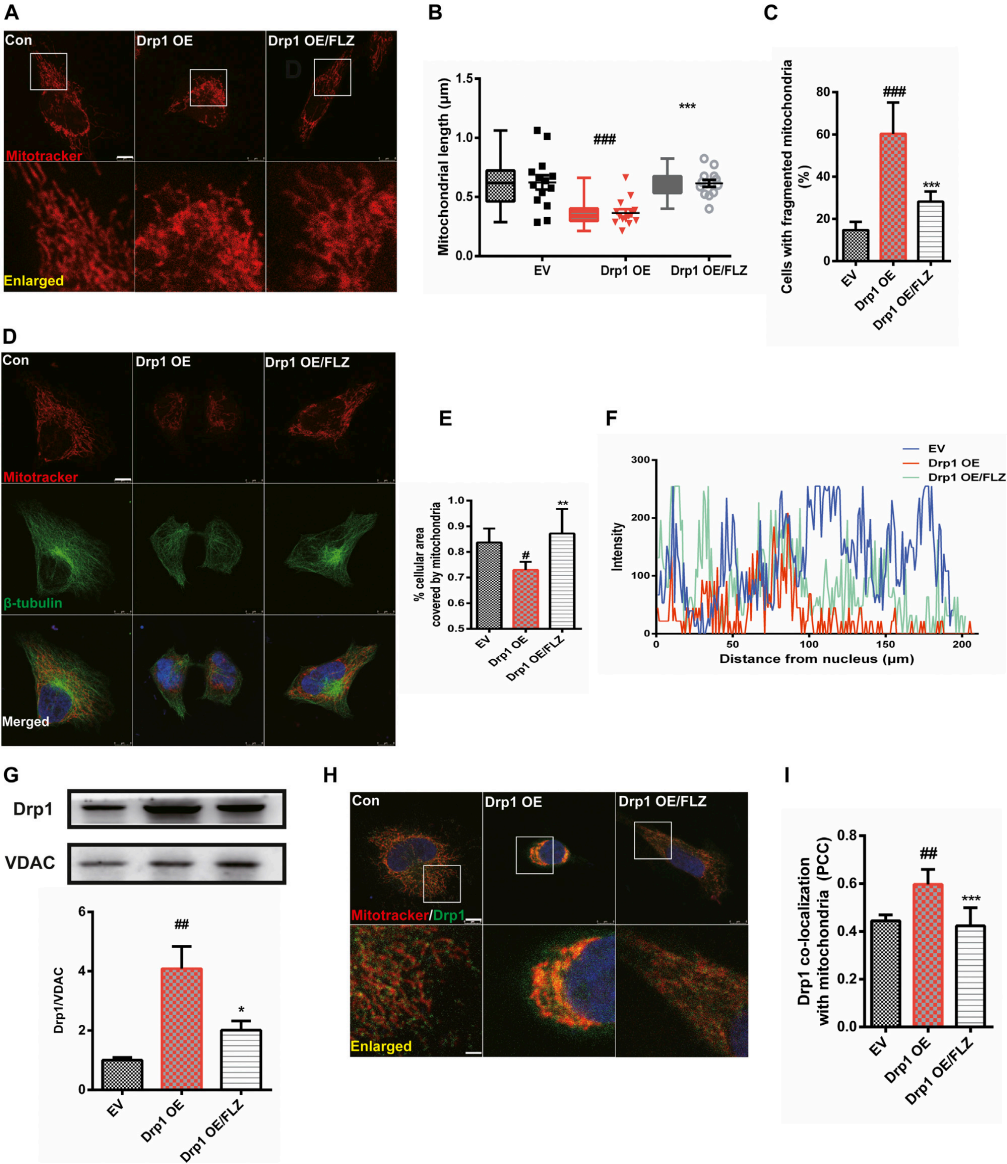
FLZ treatment reduced mitochondrial fragmentation in Drp1 overexpressed cells. Cultured Hela cells were transfected with a plasmid encoded Drp1 protein for 24 h and then the cells were incubated with FLZ for 24 h **(A)** Mitochondrial morphology was analyzed by a confocal microscope. Cells were stained with mitotracker (red). Lower panels show enlarged areas of the white boxes in the above panels (Scale bar: up: 8 μm. down: 2.5 μm). **(B)** Average mitochondrial length measured by Image-pro Plus software. Data are presented as the means ± SEM from 4 independent experiments of 20–30 random selected cells **(C)** The percentage of cells with fragmented mitochondria relative to the total number of cells was counted by an observer blinded to the experimental conditions. At least 200 cells per group were counted. **(D)** Mitochondrial distribution was analyzed by a confocal microscope. Cells were stained with mitotracker (red), *β*-tubulin (green) and DAPI (blue) (scale bar: 8 μm). **(E)** The percentage of cellular area covered by mitochondria were measured by ImageJ Software. **(F)** Shown are representative data of mitochondria (red fluorescence) distribution. **(G)** Western blot analysis of mitochondrial fractions of cells was determined by Drp1 and VDAC antibodies. VDAC was used as a loading control. **(H)** Drp1/mitochondria colocalization was determined by confocal analysis. Cells were stained with Drp1 antibody (green), mitotracker (red) and DAPI (blue). Lower panels show enlarged areas of the white boxes in the above panels (Scale bar: up: 8 μm, down: 2.5 μm). **(I)** Pearson’s coefficient per cell of (Drp1/mitochondria colocalization) was determined using Image-pro Plus software and is provided as a histogram. One-way ANOVA followed by Tukey post hoc comparisons tests were performed in all statistical analyses. ^#^ *p* < 0.05, ^##^ *p* < 0.01 and ^###^ *p* < 0.001 vs. Control group, ^*^ *p* < 0.05, ^**^ *p* < 0.01 and ^***^ *p* < 0.001 vs. Drp1 overexpression group.

We transfected the Drp1 plasmid into Hela cells to confirm the essential role of Drp1 in the process of FLZ regulation of abnormal mitochondria fission. Confocal image analysis demonstrated that the mitochondrial morphology in cells overexpressing Drp1 was punctate or fragmented and that FLZ treatment significantly alleviated the mitochondrial damage (Figures 4A,B, *p* = 0.0007 vs. MPP^+^ treated group). Furthermore, we analyzed the percentage of fragmented mitochondria and found that FLZ decreased the percentage of fragmented mitochondria in Hela cells that overexpressed Drp1 (Figure 4C *p* = 0.0004 vs. MPP^+^ treated group). Drp1 overexpression also resulted in abnormal mitochondrial distribution. Treatment with FLZ dramatically corrected these abnormal phenomena (Figures 4D,E, *p* = 0.0078 vs. MPP^+^ treated group and F). FLZ also reduced Drp1 expression (*p* = 0.0214 vs. MPP^+^ treated group) and the co-localization of Drp1 and mitochondria (*p* = 0.0009 vs. MPP^+^ treated group) in cells that overexpressed Drp1 (Figures 4G–I).

### FLZ Improved Mitochondrial Function Through Moderating Mitochondrial Fission.

**FIGURE 5:**
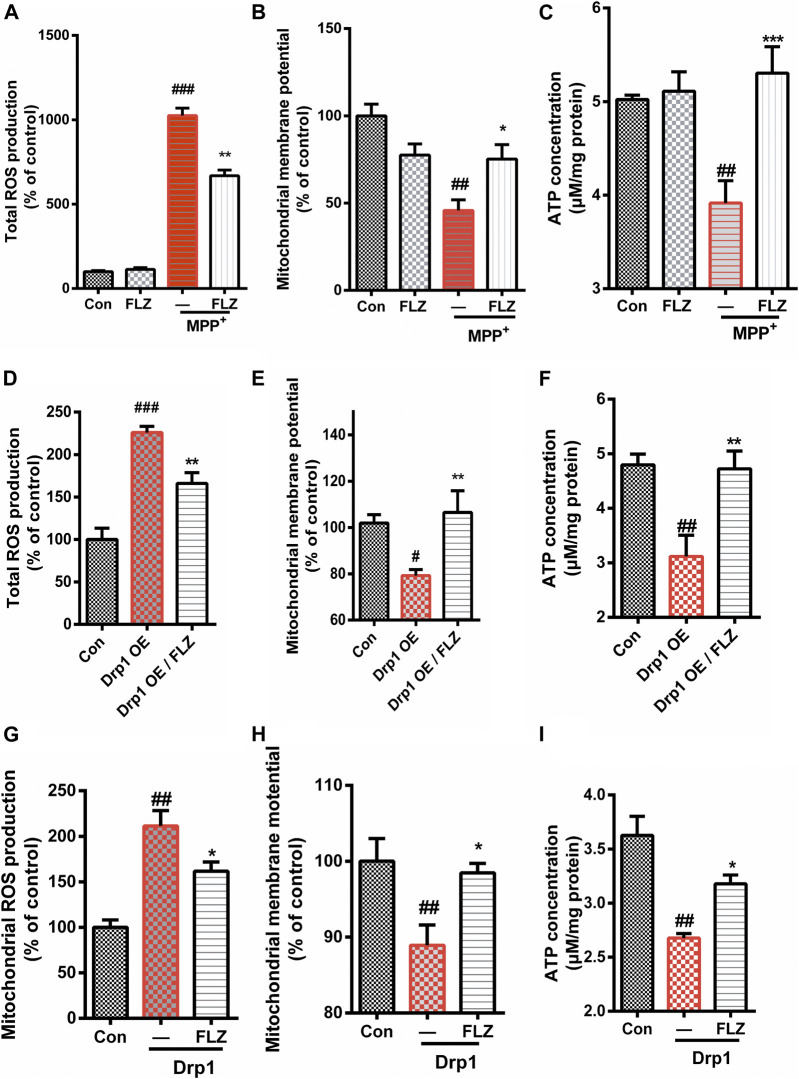
FLZ treatment reduced mitochondrial dysfunction. Cultured SH-SY5Y cells were treated with FLZ (10 μM) for 30 min followed by incubation with or without MPP^+^ (2 mM) for 6 h. Levels of **(A)** Total ROS expression **(B)** Mitochondrial membrane potential, **(C)** ATP concentration were measured. Cultured Hela cells were transfected with a plasmid encoded Drp1 protein for 24 h and then the cells were incubated with FLZ for 24 h. Levels of **(D)** Total ROS expression, **(E)** Mitochondrial membrane potential, **(F)** ATP concentration were measured. To evaluated the direct effects of activated Drp1 on the mitochondria, we incubated Drp1 recombinant protein with isolated mouse liver mitochondria. Levels of **(G)** Total ROS expression, **(H)** Mitochondrial membrane potential concentration and **(I)** ATP concentration were measured. The data of total ROS expression and mitochondrial membrane potential are presented as percentage change relative to the control group. *n* = 4∼6. One-way ANOVA followed by Tukey post hoc comparisons tests were performed in all statistical analyses. ^##^ *p* < 0.01 and ^###^ *p* < 0.001 vs. Control group, ^*^ *p* < 0.05 and ^**^ *p* < 0.01 vs. MPP^+^ treated group.

We investigated the effects of FLZ treatment on mitochondrial dysfunction caused by excessive mitochondrial fission. Treatment of SH-SY5Y cells with FLZ abolished MPP^+^-induced production of ROS (Figure 5A, *p* = 0.0042 vs. MPP^+^ treated group). We also found that FLZ treatment significantly improved mitochondrial membrane potential (*p* = 0.0391 vs. MPP^+^ treated group) and enhanced ATP production (*p* = 0.0006 vs. MPP^+^ treated group) in MPP^+^-treated SH-SY5Y cells (Figures 5B,C). In Hela cells that overexpressed Drp1, FLZ also improved mitochondrial function by reducing ROS production (*p* = 0.0041 vs. MPP^+^ treated group), increasing mitochondrial membrane potential (*p* = 0.0060 vs. MPP^+^ treated group), and promoting ATP production (*p* = 0.0049 vs. MPP^+^ treated group) (Figures 5D–F). To evaluate the direct effects of activated Drp1 on mitochondria, we incubated recombinant Drp1 protein with mitochondria isolated from mouse brains. Drp1 exposure significantly increased ROS production, decreased the mitochondrial membrane potential, and reduced ATP production. FLZ treatment partially negated these adverse effects (Figure 5G, *p* = 0.0110 vs. MPP^+^ treated group, H, *p* = 0.0236 vs. MPP^+^ treated group, and I *p* = 0.0421 vs. MPP^+^ treated group). These data indicated that FLZ reduced mitochondrial dysfunction through the regulation of Drp1.

### FLZ Treatment Reduced Neurite Degeneration and Mitochondrial Fragmentation in Primary Dopaminergic Neurons Exposed to MPP^+^

**FIGURE 6:**
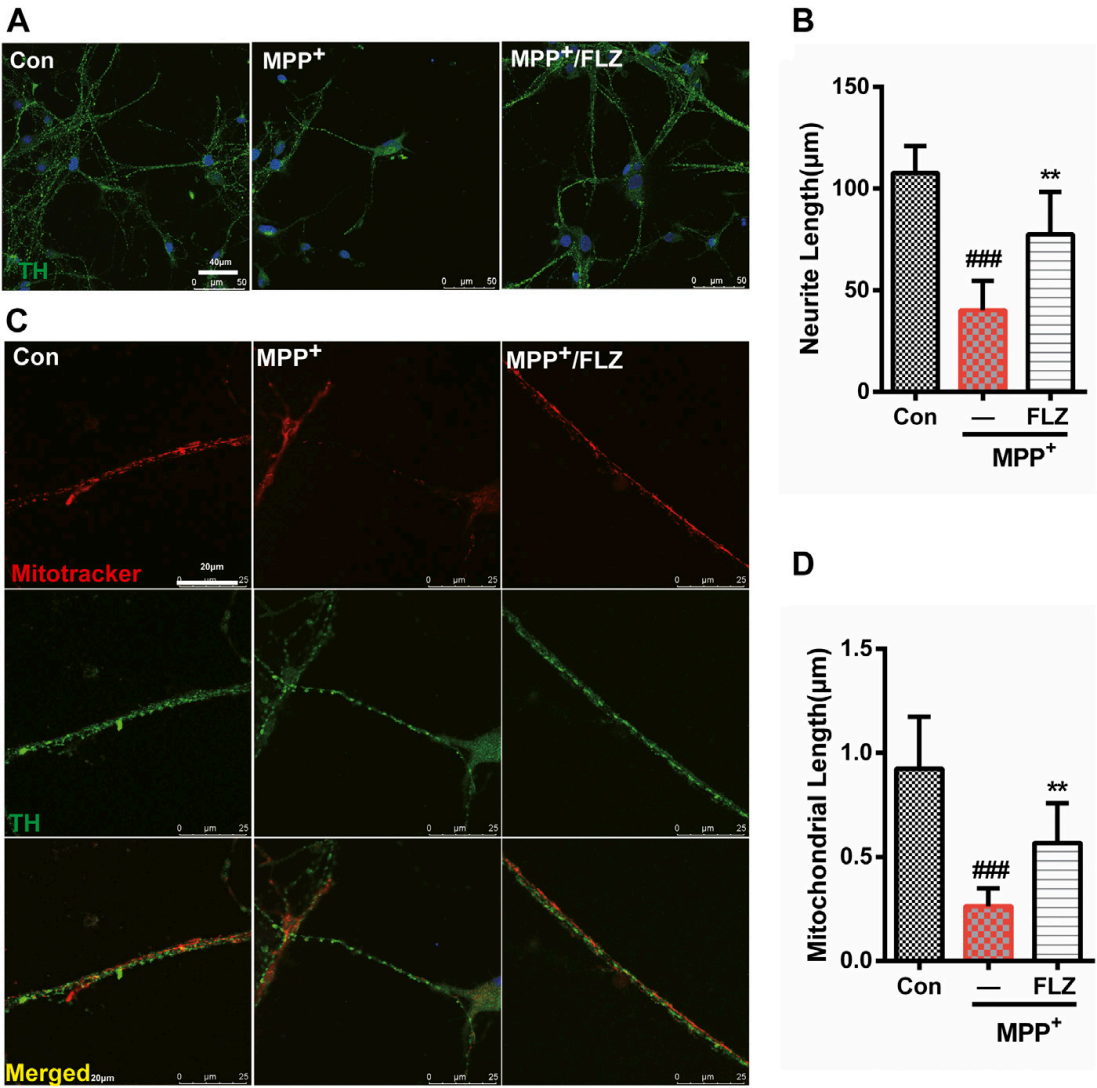
FLZ treatment reduced neurite degeneration and mitochondrial fragmentation in primary dopaminergic neurons induced by MPP^+^ Primary rat dopaminergic neurons were treated with FLZ (10 μM) followed by incubated with or without MPP^+^ (10 μM) for 24 h **(A)** Cells were stained with TH antibody (green) and DAPI (blue). (Bar: 50 μm) **(B)** Neurite length of primary dopaminergic neurons were measure by Image-pro Plus software **(C)** Cells were stained with TH antibody (green) and mitotracker (red) (Bar: 20 μm). **(D)** Mitochondrial length of primary dopaminergic neurons was measure by Image-pro Plus software. One-way ANOVA followed by Tukey post hoc comparisons tests were performed in all statistical analyses. ^###^ *p* < 0.001 vs. Control group, ^*^ *p* < 0.05 and ^**^ *p* < 0.01 vs. MPP^+^ treated group.

Aberrant mitochondrial fission occurs in many neurodegenerative diseases, including PD (Bose and Beal, 2016), which suggests that dysfunctional mitochondrial dynamics may be a critical mechanism in neurodegenerative diseases. Therefore, we verified the effects of FLZ on mitochondrial fission using cultured primary dopaminergic neurons. In this study, we found that exposure to MPP^+^ substantially reduced the length of dopaminergic cell neurites and that FLZ treatment increased the neurite length for dopaminergic neurons exposed to MPP^+^ (Figures 6A,B, *p* = 0.0014 vs. MPP^+^ treated group). Consistent with these observations, FLZ treatment reduced mitochondrial fragmentation in primary dopaminergic neurons exposed to MPP^+^, as demonstrated by increased mitochondria lengths (Figures 6C,D, *p* = 0.0058 vs. MPP^+^ treated group). These data from a cell culture model of PD suggested that inhibition of Drp1-induced mitochondrial dysfunction by FLZ resulted in decreased neuronal degeneration.

### FLZ Reduced Mitochondrial Fragmentation and Protected Dopaminergic Neurons Through the Regulation of Drp1 in MPTP-Treated PD Mice.

**FIGURE 7:**
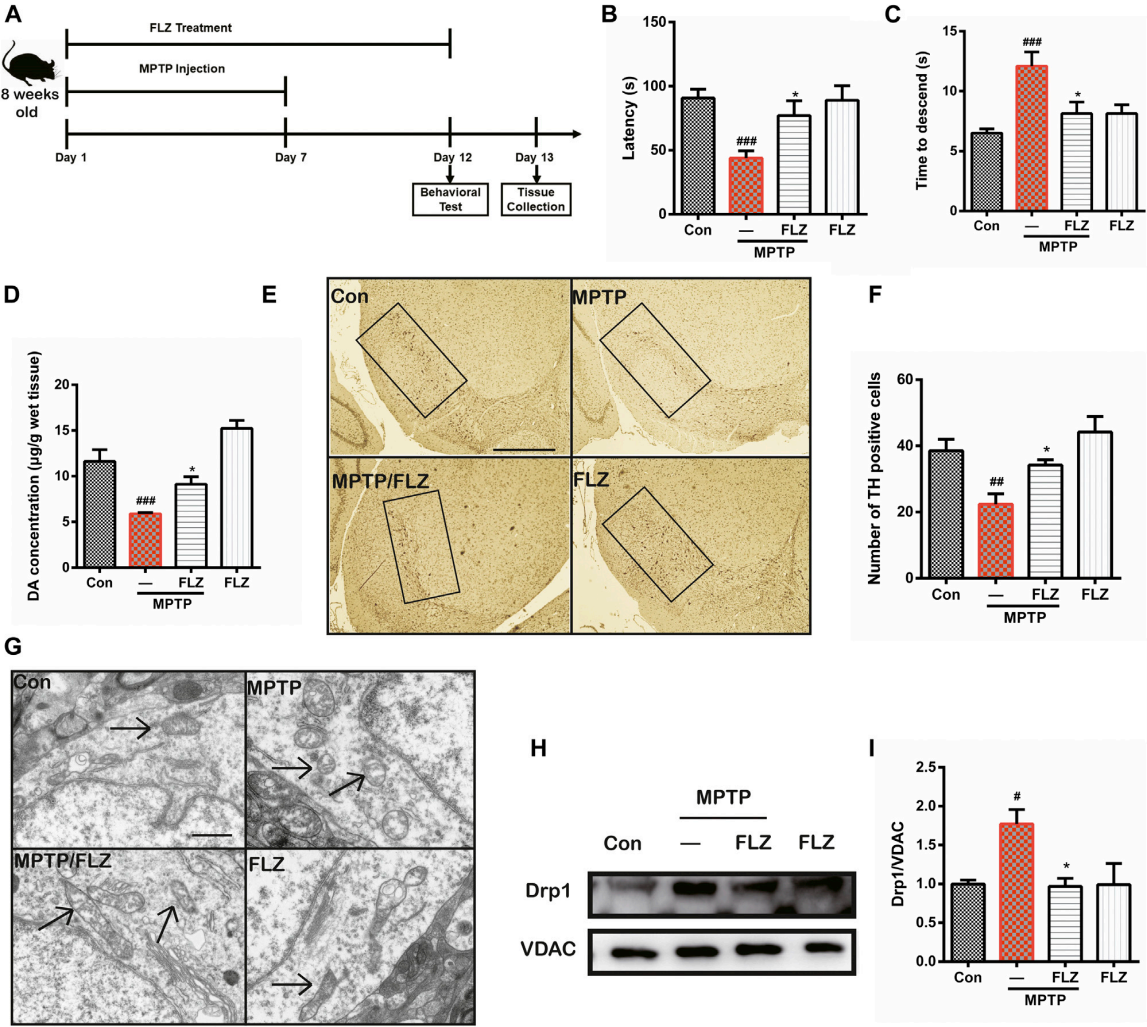
FLZ treatment reduced motor dysfunction and mitochondrial fragmentation in MPTP-treated mice **(A)** the experimental arrangement. Measurement of mice motor performance using the **(B)** rotarod test and **(C)** pole test **(D)** DA concentration was measured by HPLC. One-way ANOVA followed by Bonferroni’s post hoc comparisons tests were performed in statistical analyses. **(E, F)** The number of TH-positive neurons in SNpc was counted and provided in a histogram **(G)** The morphology of mitochondria was measured by electron microscope. **(H, I)** Western blot analysis of mitochondrial fractions of tissue was determined by Drp1 and VDAC antibodies. VDAC was used as a loading control. One-way ANOVA followed by Tukey post hoc comparisons tests were performed in all statistical analyses. ^###^ *p* < 0.001, ^##^ *p* < 0.01 and ^#^ *p* < 0.05 vs. Control group, ^*^ *p* < 0.05 vs. MPP^+^ treated group.

We conducted *in vivo* mouse experiments to validate the effects of FLZ treatment on the role of Drp1 in regulating mitochondria fission. As seen in Figure 7A, mice were treated with FLZ 30 min before each MPTP hydrochloride injection for seven consecutive days. From days 8–12, the mice continued to be treated with FLZ but did not receive any MPTP treatment. Behavior testing revealed that the MPTP-treated mice spent less time on the rod and more time moving down the pole), and FLZ treatment significantly improved the motor behavioral performance of the MPTP-treated mice (Figure 7B *, p* = 0.0237 vs. MPP^+^ treated group and Figure 7C, *p* = 0.0308 vs. MPP^+^ treated group). FLZ also improved dopaminergic neuron function, as indicated by increased concentrations of dopamine in the striatum (Figure 7D, *p* = 0.0376 vs. MPP^+^ treated group), and enhanced TH-positive neurons in the SNpc in MPTP-treated mice (Figures 7E,F, *p* = 0.0498 vs. MPP^+^ treated group).

Furthermore, observation of mitochondrial ultrastructure using electron microscopy showed that mitochondria in MPTP-treated mice exhibited a punctate appearance, while FLZ treatment substantially increased the length of the mitochondria (Figure 7G). Western blot analysis verified our observation that FLZ treatment decreased Drp1 translocation from the cytosol to the mitochondrial surface in the MPTP-induced PD mouse model (Figure 7H, *p* = 0.0215 vs. MPP^+^ treated group and I). All these data supported our hypothesis that FLZ reduced mitochondrial fragmentation and protected dopaminergic neurons in MPTP-treated mice through the regulation of Drp1.

## Discussion

In this study, we investigated the role and underlying mechanisms by which Drp1 regulated mitochondrial function and the neuroprotective actions of FLZ in PD. The data revealed that FLZ markedly diminished mitochondrial fragmentation and abnormal distribution in MPP^+^-challenged dopaminergic neurons. Drp1 played an essential role in the process of FLZ modulation of mitochondrial dynamics. FLZ attenuated Drp1 activity in MPP^+^-treated SH-SY5Y cells and Hela cells that overexpressed Drp1, which improved mitochondrial function. FLZ treatment also alleviated motor dysfunction, protected dopaminergic neurons, and reduced mitochondrial fragmentation through the regulation of Drp1 in MPTP-treated mice. The present data indicated that FLZ alleviated mitochondrial fragmentation by inhibiting Drp1 activation and protected dopaminergic neurons, which might be a fundamental mechanism of FLZ in the treatment of PD.

Mitochondrial fragmentation has been observed *in vivo* and *in vitro* PD models, and abnormal mitochondrial accumulation in the perinuclear area of cells also has been reported (Chuang et al., 2016; Trevisan et al., 2018; Zhou et al., 2018), These findings indicate that mitochondrial dysregulation plays an important role in the course of PD. Mitochondrial dysfunction results in decreased ATP production, increased free radical production, imbalances in calcium homeostasis, mitochondrial DNA mutations, and abnormal protein deposition, all of which affect the function of neurons and glial cells (Lane et al., 2015; Golpich et al., 2017), and lead to neuronal loss (Burté et al., 2015). Mitochondria are the central endogenous sources of cellular ROS, and increased ROS production reflects respiratory chain damage or dysfunction (Cid-Castro et al., 2018). ROS production promotes inflammation, which also can lead to neuronal apoptosis (Kim et al., 2016). Our study showed that FLZ treatment reduced ROS production in SH-SY5Y cells treated with MPP^+^. These data are consistent with recent studies demonstrating that mitochondrial fragmentation mediated by fission is a necessary component to ROS overproduction and that cells with fragmented mitochondria demonstrate enhanced ROS production (Wang et al., 2009; Wang et al., 2008).

Cells need energy to maintain their viability, growth, and normal function. The main source of chemical energy in cells is ATP, and oxidative phosphorylation in mitochondria is the primary way cells produce ATP (Mookerjee et al., 2017). Consistent with previous studies, we found that MPP^+^ induced a decrease in intracellular ATP levels (Wang et al., 2011b) and exposure to FLZ increased ATP production. These results indicated that the neuroprotective effects of FLZ on PD are closely related to improved mitochondrial function.

Mitochondrial dynamics composed of mitochondrial fission and fusion play a vital role in regulating mitochondrial morphology, distribution, and transport (Friedman and Nunnari, 2014; Zemirli et al., 2018). In the PD model, excessive mitochondrial fission induced by exposure to neurotoxins and mutations in PD-related genes is thought to result in mitochondrial autophagy, which eventually leads to degenerative lesions in dopaminergic neurons (Lee et al., 2010; Franco-Iborra et al., 2016). Thus, inhibition of excessive mitochondrial fission and eliminating damaged mitochondria due to excessive mitochondrial fission might be an important strategy to reduce or reverse the pathogenesis of PD. Our results demonstrated that FLZ significantly reduced mitochondrial fragmentation and alleviated aberrant mitochondrial distribution in MPP^+^-treated SH-SY5Y cells and cells overexpressing Drp1. These observations were further validated by our *in vivo* results that revealed increased mitochondrial lengths after treatment with FLZ. The primary result of FLZ treatment after MPP^+^ exposure was observed for Drp1 protein. Thus, these data indicated that FLZ treatment might diminish mitochondrial fragmentation by inhibiting excessive mitochondrial fission through the regulation of Drp1 protein.

Translocation of Drp1 to mitochondria from the cytosol is a hallmark of mitochondrial fission. We observed that FLZ substantially decreased the co-localization of Drp1 and mitochondria (Figures 3A–C). Drp1 activity was essential in mediating mitochondrial dynamics. It has been reported that the posttranslational phosphorylation of Drp1 has important roles in mitochondrial dynamics and cell fate determination (Qi et al., 2011; Jahani-Asl et al., 2015). For example, PKA-mediated Drp1 phosphorylation at Ser637/656 attenuated the GTPase activity of Drp1 and promoted cell survival. However, phosphorylation of Drp1 at Ser616 induced its translocation to mitochondria, increased mitochondrial fragmentation, and promoted cell death (Bo et al., 2018).

In this study, we measured the phosphorylation level of Drp1 at Ser616. We found that FLZ decreased the upregulation of Drp1 phosphorylation at S616 with exposure to MPP^+^. In addition, FLZ reduced the GTPase activity of Drp1 in SH-SY5Y cells (Figure 3D). We suggested that FLZ could directly bind to Drp1 because Drp1 could decrease the GTPase of Drp1 without mitochondrial adaptors being present (Figure 3E). We transfected Hela cells with Drp1 plasmids to prove the hypothesis that FLZ reduced mitochondrial fragmentation via regulation of Drp1 activation. We found that FLZ treatment partially prevented the impairment of mitochondrial fission and abnormal mitochondrial distribution induced by Drp1 overexpression (Figure 4). Thus, these effects might be attributed to the reduction of the association of Drp1 with mitochondria when exposed to FLZ. Also, we evaluated the effect of FLZ on MPTP-treatment in an animal model. We observed that FLZ had a neuroprotective effect on primary dopaminergic neurons exposed to MPP^+^. Consistent with our findings *in vitro*, FLZ treatment reduced mitochondrial fragmentation in cells from MPTP-treated mice, alleviated motor dysfunction, and protected dopaminergic neurons. These results suggested that the regulation of mitochondrial dynamics by Drp1 plays a critical role in PD pathogenesis. Inhibiting Drp1 might be a useful treatment for neurodegenerative diseases that exhibit impaired mitochondrial dynamics.

Procedures designed to limit mitochondrial damage and ensure cell integrity by inhibiting mitochondrial fission damage are a new therapeutic strategy. The foundation of mitochondrial fission is Drp1. Researchers have designed small molecules that target Drp1. For example, the first discovered inhibitor of Drp1, Mdivi-1, prevents Drp1 self-assembly into rings and its association with mitochondria (Lackner and Nunnari, 2010), The Drp1 oligomerization process is essential for GTPase activity (Cassidy-Stone et al., 2008). Another Drp1 inhibitor, P110, binds with Drp1 directly and inhibited Drp1 GTPase activity in a PD cell model (Qi et al., 2013). These small molecules demonstrated neuroprotective effects via inhibition of Drp1 activity. According to our molecular docking analysis, we concluded that FLZ might inhibit Drp1 activity through directly binding to the active site of Drp1.

Overall, our studies suggested that Drp1 inhibition is a promising therapy to reduce excessive mitochondrial fission, increase mitochondrial fusion, and maintain mitochondrial function in neurons affected by PD. Our findings support a new therapeutic strategy focused on Drp1 as a target for PD treatment. Given that the balance of fission and fusion is important for normal mitochondrial function, clinicians need to be vigilant ensuring that the use of Drp1 inhibitors remains restricted to correct abnormal mitochondrial fission until further evidence is established. Therefore, inhibition of Drp1 by FLZ might be a useful treatment for neurodegeneration diseases associated with impaired mitochondrial dynamics.

## Data Availability

The original contributions presented in the study are included in the article/Supplementary Material, further inquiries can be directed to the corresponding author.
